# Near‐Infrared Emission Perovskites for Multifunctional Bioimaging

**DOI:** 10.1002/smsc.202500033

**Published:** 2025-02-21

**Authors:** Tianchi Wang, Jiabo Sun, Zhaowei Teng, Shuyi Yao, Junheng Yuan, Lulu Han, Dedan Mu, Hao Song, Xue Yu, Xuhui Xu

**Affiliations:** ^1^ The Central Laboratory and Department of Orthopedic The Second Affiliated Hospital of Kunming Medical University Kunming Yunnan 650106 P. R. China; ^2^ Faculty of Materials Science and Engineering Key Laboratory of Advanced Materials of Yunnan Province Kunming University of Science and Technology Kunming Yunnan 650093 P. R. China; ^3^ School of Mechanical Engineering Chengdu University Chengdu Sichuan 610106 P. R. China

**Keywords:** near‐infrared emissions, perovskites, scintillators, X‐ray imagings

## Abstract

Bioimaging with remarkable noninvasive nature, ultrahigh resolution and sensitivity allows detection of pathologies of bones, organs, and tissues. Nevertheless, the achievement of more complete information in vivo is challenged by the necessity of multiple photodetectors with diverse response ranges. Herein, a multifunctional bioimaging with Cs_2_AgInCl_6_:Yb^3+^ perovskites via a single InGaAs detector for superior tissue presentation is realized in this work. Co‐incorporation of foreign dopant contributes to alterations of local structural symmetry of the Cs_2_AgInCl_6_ host, disruption of parity‐forbidden transitions, and reduction in electron–phonon coupling strength, thereby boosting the near‐infrared (NIR) intensity by 40‐fold of the corresponding perovskites drastically. Moreover, an X‐ray excited NIR light output is 2.83 times that of commercial Bi_4_Ge_3_O_12_ scintillators. Thanks to the efficient NIR emission, the versatile perovskites film endows a multifunctional bioimaging with detailed information of biological tissue in vivo, which fundamentally offers viable avenues for promoting bioimaging technology with integrated access of tissue presentation.

## Graphical abstract

Currently, the acquisition of information inside living organisms is highly dependent on a variety of photodetectors with different response ranges, for which a near‐infrared‐emitting perovskite material has been designed that exhibits excellent performance under both UV and X‐ray irradiation, enabling multimodal imaging of biological tissues with only one InGaAs detector.

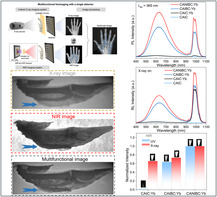

## 1 Introduction

Biological image techniques primarily utilize optical,^[^ ^1^ ^]^ magnetic resonance,^[^ ^2^ ^]^ radiological,^[^ ^3^ ^]^ ultrasound,^[^ ^4^ ^]^ and computed tomography,^[^ ^5^ ^]^ to capture microscopic tissues images of living organisms. Therefore, bioimaging reveals the intricate composition and functionality of biological interiors,^[^ ^6^ ^]^ being indispensable visualization tools and means in modern life science research and medical practice. Notably, X‐ray^[^ ^3^ ^]^ and near‐infrared (NIR)^[^ ^7^ ^]^ imaging stand out for their noninvasive nature, high sensitivity, and specificity. It fundamentally mitigates patient discomfort and surgical risks while detecting subtle lesions undetectable by conventional methods, herein, has been widely applied in early diagnosis, disease monitoring, and treatment efficacy assessment.

In fact, X‐ray imaging exploits the fact that tissues with higher density absorb more X‐ray photons, such as bones, while softer tissues like blood vessels, muscles, and fat absorb less energy; hence, high‐contrast black‐and‐white grayscale images could be obtained.^[^ ^8^ ^]^ NIR imaging offers the capability to visualize blood vessels, lymphatic vessels, and specific low‐density tissues due to the biological penetration of the NIR characteristics.^[^ ^9^ ^]^ It reveals pathological regions that even may be overlooked by X‐ray imaging.^[^ ^10^ ^]^ Hence, the two imaging modalities could be complementary for medical diagnosis and treatment. Unfortunately, due to the limited response range of photodetectors, indirect X‐ray and NIR imaging typically require different detectors, tailored to the visible (400–780 nm) and NIR (780–1700 nm) spectral regions respectively. Hence, image overlay processing employed to obtain comprehensive information of organisms^[^ ^11^ ^]^ often faces the dilemma that images acquired from different detectors suffer from resolution mismatch, distinct dynamic ranges, signal‐to‐noise ratios differences, and difficulties in image registration.^[^ ^12^ ^]^ Therefore, bioimaging with a single photodetector is of great significance not only for getting physical information of the patients more conveniently and accurately but also for promoting advanced medical imaging technology.

**Figure 1:**
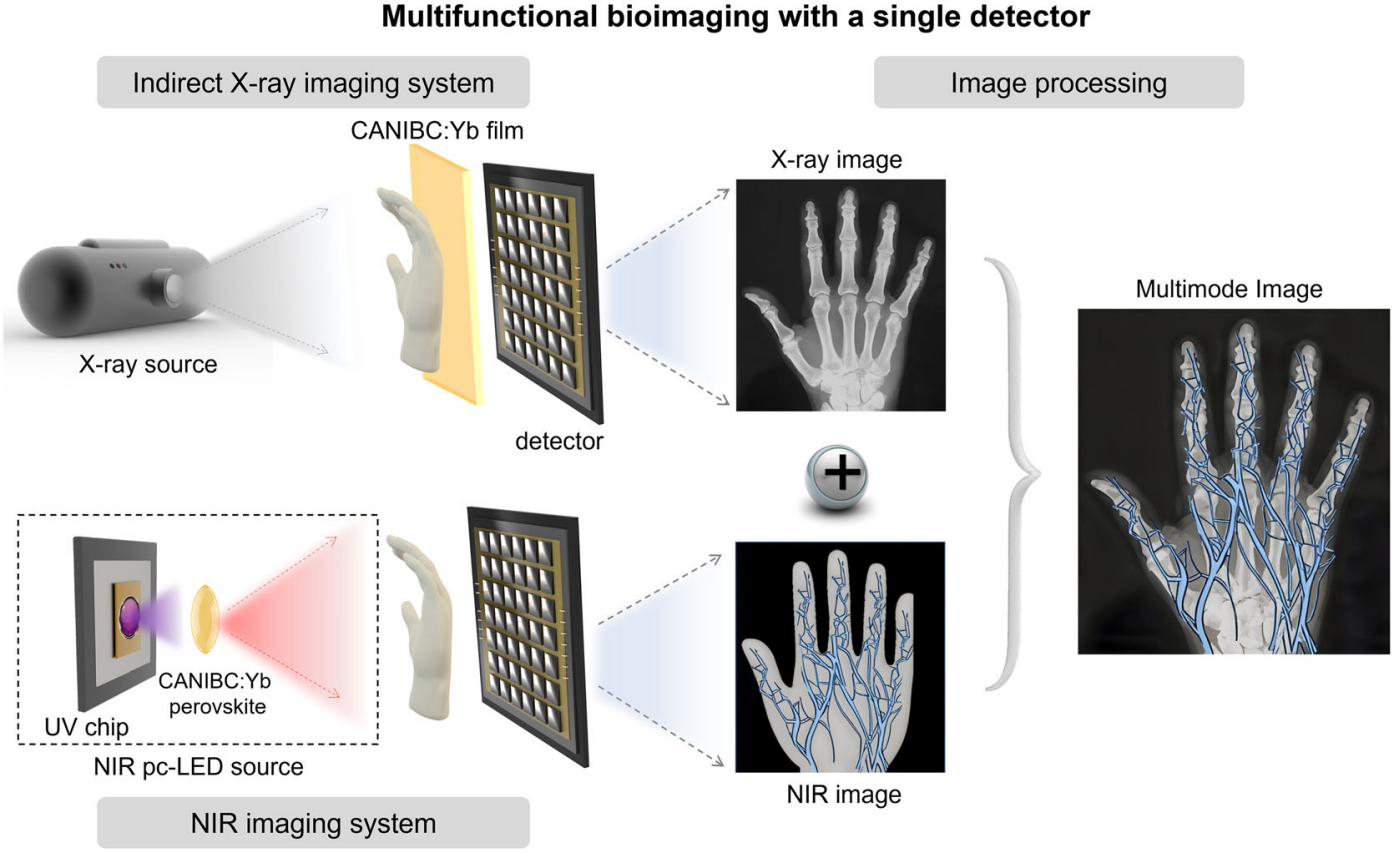
Schematic illustration of X‐ray and NIR imaging was achieved with a single InGaAs detector.

In this work, Na^+^‐ and Bi^3+^‐co‐alloyed Cs_2_AgInCl_6_:Yb^3+^ (CAIC:Yb) perovskites that exhibit remarkable NIR luminescence under X‐ray or UV light excitation were explored. It enables the achievement of multifunctional imaging solely with an InGaAs detector for the response range spans from 0.9 to 1.7 μm (**Figure** **1**). Remarkably, the NIR emission intensity of the explored perovskites under X‐ray irradiation and ultraviolet excitation is significantly enhanced with the crystal structure regulation. Herein, leveraging the robust anti‐interference capability of NIR in vivo, the corresponding perovskites enable a high‐quality multifunctional imaging even operated in an ambient environment with strong external light crosstalk. Accordingly, this work presents a straightforward and feasible research avenue for multifunctional bioimaging, prompting an advanced approach for in vivo tissues information acquisition.

## 2 Results and Discussion

Single‐crystal samples of Cs_2_AgInCl_6_ (CAIC), CAIC:Yb, Cs_2_Ag_0.6_Na_0.4_InCl_6_:Yb^3+^ (CANIC:Yb), and Cs_2_Ag_0.6_Na_0.4_In_0.85_Bi_0.15_Cl_6_:Yb^3+^ (CANIBC:Yb) have been prepared by a modified hydrothermal reaction (Supporting Information). The powder X‐ray diffraction (XRD) pattern in Figure S1, Supporting Information, shows that both the doped and undoped crystal samples have the cubic structure of CAIC (ICSD No. 244519), no additional impurity peaks are observed, and the diffraction peaks are shifted in a small range due to doping. The actual doping concentrations of Yb^3+^ are listed in Table S1, Supporting Information.

Scanning electron microscopy images show micron‐sized crystals with polygonal shapes for the as‐obtained crystal samples doped with Yb^3+^, Bi^3+^, and Na^+^ (Figure S2, Supporting Information). Moreover, energy‐dispersive X‐ray mapping indicates the high quality of the synthesized corresponding crystal samples. As shown in Figure S3, Supporting Information, the energy spectrum analysis of the CANIBC:Yb crystal indicates that the elements of Cs, Ag, Na, In, Bi, and Cl are generally consistent with their chemical composition. Furthermore, the content of Yb element is determined to be 1.21%, which is identical to the results of inductively coupled plasmamass spectrometry (Table S2, Supporting Information). X‐ray photoelectron spectroscopy analysis of the CAIC, CAIC:Yb, CAIBC:Yb, and CANIBC:Yb crystal samples features the signal peaks of the corresponding elements in Figure S4, Supporting Information.

**Figure 2:**
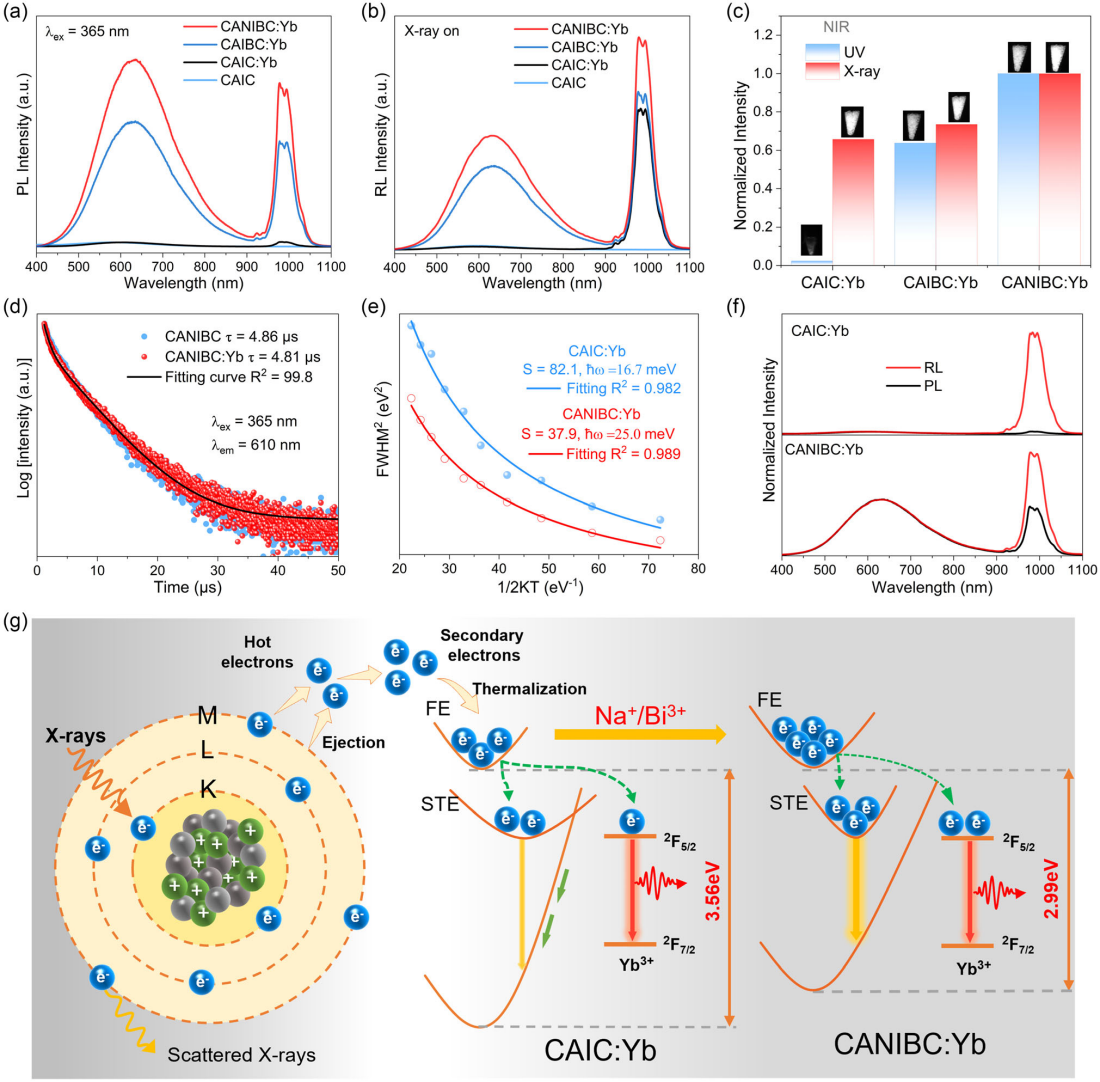
a) PL, b) RL spectra, and c) normalized intensity ratios of NIR emission of the CAIC:Yb, CAIBC:Yb and CANIBC:Yb crystal samples, respectively. The inset shows the PL and RL NIR photograph of the corresponding crystal samples captured with InGaAs detector. d) PL decay curves (*λ*_ex_ = 365 nm, *λ*_em_ = 600 nm), e) FWHM of PL versus temperature, f) PL and RL spectra of CANIBC and CANIBC:Yb, and g) schematic illustration of the scintillation process.

Furthermore, the effect of co‐alloying Na^+^ and Bi^3+^ on the luminescent properties of CAIC:Yb was investigated under UV excitation and X‐ray irradiation, respectively (**Figure** **2**a,b). Under X‐ray and UV light excitation, the CAIC crystal sample displays a faint broadband self‐trapped exciton (STE) emission centered at 593 nm, which is in agreement with previously published reports.^[^ ^13^ ^]^ It is noteworthy that the intensity of the STE emission is enhanced by about 50‐fold for the incorporation of Na^+^ and Bi^3+^ (Figure S5, Supporting Information). Moreover, CAIC:Yb shows typical NIR emission of ^2^F_5/2_–^2^F_7/2_ transition^[^ ^14^ ^]^ originated from Yb^3+^, and the NIR spectra under UV and X‐ray irradiation are depicted in Figure 2c. CAIC:Yb crystal sample exhibits weak NIR luminescence (PL quantum yield [PLQY] < 1%) under UV light excitation, with the NIR emission intensity is enhanced by ≈40 times through the alloying with Na^+^ and Bi^3+^. Conversely, the CAIC:Yb crystal sample exhibits stronger NIR emission intensity for the incorporation of Bi^3+^ with the absence of Na^+^ under X‐ray irradiation. Notably, the difference in the NIR performance of the CAIC:Yb crystal samples under UV and X‐ray irradiation is recorded unambiguously with a InGaAs detector (inset of Figure 2c).

The absorption spectra of CAIC and CAIC:Yb crystal samples exhibit strong absorption at ≈320 nm and relatively weaker absorption at ≈390 nm (Figure S6a, Supporting Information). Notably, the crystal sample doped with Bi^3+^ exhibits a marked enhancement of the absorption band centered at ≈390 nm. As shown in Figure S6b, Supporting Information, the optical bandgaps of the CAIC and CANIBC:Yb crystal samples are determined to be 3.53 and 2.99 eV by Tauc plots, respectively. Moreover, density‐functional theory calculations based on the generalized gradient approximation of the Perdew–Burke–Ernzerhof generalization were carried out (Figure S7, Supporting Information). The calculation results indicate that the bandgap of CAIC is reduced from 2.078 to be 1.948 eV for the incorporation of Na^+^ and Bi^3+^, which is consistent with the observed trend of the experimental results. Furthermore, by monitoring the photoluminescence (PL) and PL excitation spectra of the STE emission (Figure S8a, Supporting Information), it is evident that the introduction of Na^+^ and Bi^3+^ leads to the emergence of an excitation band at 370 nm for the significantly enhanced absorption of the excitation intensity. Moreover, the PL decay curves of CAIC:Yb and CANIBC:Yb suggest that the introduction of Na^+^ and Bi^3+^ prolongs the STE luminescence decay lifetime, effectively inhibiting the non‐radiative relaxation process (Figure S8b, Supporting Information).

The doping of Yb^3+^ into double perovskite lattices has been demonstrated to generate NIR emission,^[^ ^15^ ^]^ yet the fundamental energy transfer mechanism underlying this process remains elusive. Currently, there exists two primary modes of energy transfer from double perovskite materials to lanthanide‐doped ions (Ln^3+^): one entails energy transfer from STE to Ln^3+^,^[13a]^ and the other involves energy transfer from free excitons to Ln^3+^.^[^ ^16^ ^]^ The fundamental distinction between these two mechanisms stems from the fact that the former results in the luminescence quenching of STE, whereas the latter does not. As depicted in Figure 2a, the absence of quenching of the STE emission for all Yb^3+^‐doped crystal samples indicates that no energy transfer takes place between STE and Yb^3+^. This conclusion is further verified by the fact that the STE luminescence decay lifetime of CANIBC crystal sample shows insignificant changes after Yb^3+^ doping (Figure 2d).

To elucidate the photophysical mechanisms of CAIC:Yb and CANIBC:Yb, temperature‐dependent emission spectra were further recorded (Figure S9, Supporting Information). The temperature‐induced STE quenching tendency of CANIBC:Yb is suppressed after alloying with Na^+^ and Bi^3+^ (Figure S10, Supporting Information). The exciton binding energies (*E*_b_) of CAIC:Yb and CANIBC:Yb were calculated by fitting the Arrhenius equation.^[^ ^17^ ^]^

(1) $$I\left(T\right)=\frac{I_{0}}{1+Ae^{-E_{b}/k_{B}T}}$$

where *I*_0_ is the low‐temperature PL intensity and *k*_B_ is the Boltzmann constant. The fitting results indicate that the *E*_b_ values of CAIC:Yb and CANIBC:Yb are determined to be 92.6 and 167.8 meV, respectively (Figure S11, Supporting Information). Hence, the remarkable increase of *E*_b_ implies that Na^+^ and Bi^3+^ co‐alloying can effectively reduce recombination loss and thermal quenching of STE.^[^ ^18^ ^]^

The Huang–Rhys factor (*S*) can be used to assess trends in STE formation. From the perspective of photophysics, the *S* is associated with the displacement of the equilibrium positions of the nuclei upon exciting the chromophore, which reflects the strength of the electron–phonon coupling. The *S* value can be derived from temperature‐dependent full width at half maximum (FWHM) using equation.^[^ ^19^ ^]^

(2) $$\text{FWHM}=2.36\sqrt{S}\hbar \omega_{\text{phonon}}\sqrt{\text{cot}h\frac{\hbar \omega_{\text{phonon}}}{2k_{B}T}}$$

where *ħ* represents the reduced Planck constant, *ω*_phonon_ is phonon frequency, *T* is temperature, and *k*_B_ is Boltzmann constant. The *S* and *ħω* are calculated to be 82.1 and 16.7 meV for the CAIC:Yb and 37.9 and 25.0 meV for the CANIBC:Yb crystal sample (Figure 2e), respectively. Generally, the stronger the electron–phonon coupling, the larger the lattice relaxation.^[^ ^20^ ^]^ It indicates that carriers in the excited state undergo non‐radiative recombination back to the ground state by thermally stimulated crossover, reducing STE emission intensity significantly.^[^ ^21^ ^]^ As shown in Table S3, Supporting Information, the *S* value of CAIC:Yb is as high as 82.1, which indicates a strong electron–phonon coupling effect, resulting in significantly reduced STE emission efficiency.^[13b]^ The *S* value of CANIBC:Yb is reduced to be 37.9, effectively inhibiting the generation of non‐radiative recombination channels and thereby improving the STE emission efficiency.

Furthermore, Raman spectra were measured under excitation by a 532 nm laser diode (Figure S12, Supporting Information). As a result, the doping of Na^+^ and Bi^3+^ intensifies the local distortion and the localization of the [AgCl_6_]^5−^ octahedron, subsequently reducing the electronic dimensionality of CAIC:Yb. The modification breaks the parity‐forbidden transition and effectively enhances the absorption, excitation, and optical properties of the corresponding crystal samples. Accordingly, a photophysical mechanism elucidating the impact of Na^+^ and Bi^3+^ co‐doping on the optical properties of CAIC:Yb is proposed (Figure S13, Supporting Information).

Moreover, a significant difference in the luminescence intensity of Yb^3+^ in CAIC:Yb crystal samples under UV and X‐ray irradiation is observed, we conducted a comparative analysis of their PL and radioluminescence (RL) spectra. As depicted in Figure 2f, the peak shapes and positions of the RL and PL spectra are identical, indicating that both luminescence processes originate from the same radiative recombination pathway.^[^ ^22^ ^]^ During the RL process, first, the heavy atoms in the double perovskite efficiently absorb radiation energy, subsequently triggering the photoelectric effect and inelastic Compton scattering mechanisms, rapidly releasing a large number of hot electrons.^[^ ^23^ ^]^ Immediately thereafter, these hot electrons undergo a thermalization process within an extremely short period, rapidly transitioning to the conduction band, and ultimately being primarily captured by the luminescence centers, completing the scintillation process. The fundamental difference between this process and UV excitation lies in the fact that RL does not undergo electron transitions from the valence band maximum to the conduction band minimum, thus avoiding the effects of parity‐forbidden transitions. As a result, CAIC:Yb generates a substantial number of free excitons under X‐ray excitation. The inherently strong electron–phonon coupling effect in the CAIC:Yb crystal sample induces non‐radiative relaxation channels, resulting in extremely weak STE emission intensity, which is consistent with the observations in the PL spectrum. However, it is crucial to note that the luminescence of Yb^3+^ is hardly affected by the electron–phonon coupling effect. Consequently, when a significant number of free excitons reach the energy levels of Yb^3+^, efficient luminescence is produced. Therefore, when the CAIC:Yb crystal sample is excited by X‐ray, the RL spectrum exhibits weak STE emission alongside intense Yb^3+^ emission phenomena.

Furthermore, light output (LO) is deemed as the ability of scintillators to convert X‐ray into low‐energy photons. In theory, LO can be described by Equation (3).^[^ ^24^ ^]^

(3) $$\text{LO}=E/\beta E_{g}\times S_{1}Q$$

where *E* represents the energy of the incident X‐ray photons, and *E*_g_ denotes the bandgap of the scintillator. *β*, indicating the average energy required to generate one thermalized electron–hole pair, is a phenomenological parameter that usually ranges between 2 and 3. Therefore, the LO is jointly determined by the efficiency of hot electron energy transfer (*S*_1_) and the PLQY (*Q*). By co‐alloying with Na^+^ and Bi^3+^, the CAIC:Yb crystal sample effectively mitigates the electron–phonon coupling effect and suppresses non‐radiative recombination, leading to significant enhancement in PLQY. A high PLQY ensures that once electrons are transferred to the recombination centers, the ultimate radiative emission is highly efficient. Given the large atomic number of Bi^3+^, the introduction of Bi^3+^ as a partial substitution for In^3+^ in the lattice can effectively enhance the absorption capacity for X‐rays. Additionally, the large ionic radius of Bi^3+^ results in a smaller distribution radius of surrounding hot electrons, favoring the process of *S*_1_.^[23b]^ Consequently, with the co‐alloying of Na^+^ and Bi^3+^, the *S*_1_ and *Q* factors of the CAIC:Yb crystal samples are optimized, leading to an improved LO. Accordingly, a schematic illustration of the scintillation process for CAIC:Yb and CANIBC:Yb is proposed (Figure 2g).

Subsequently, we prepared CAIC crystal samples doped with different Ln^3+^ (Figure S14, Supporting Information). By normalizing the STE emission peak, we observed that the RL intensity of Ln^3+^ for CAIC:Ln (Ln = Tm^3+^, Er^3+^, Nd^3+^, and Ho^3+^) is significantly stronger than its PL intensity, identical to the observations of CAIC:Yb (Figure S15, Supporting Information). It indicates that the notable discrepancy in Ln^3+^ luminescence intensity under UV and X‐ray excitation is a universal phenomenon in Ln^3+^‐doped CAIC materials.

**Figure 3:**
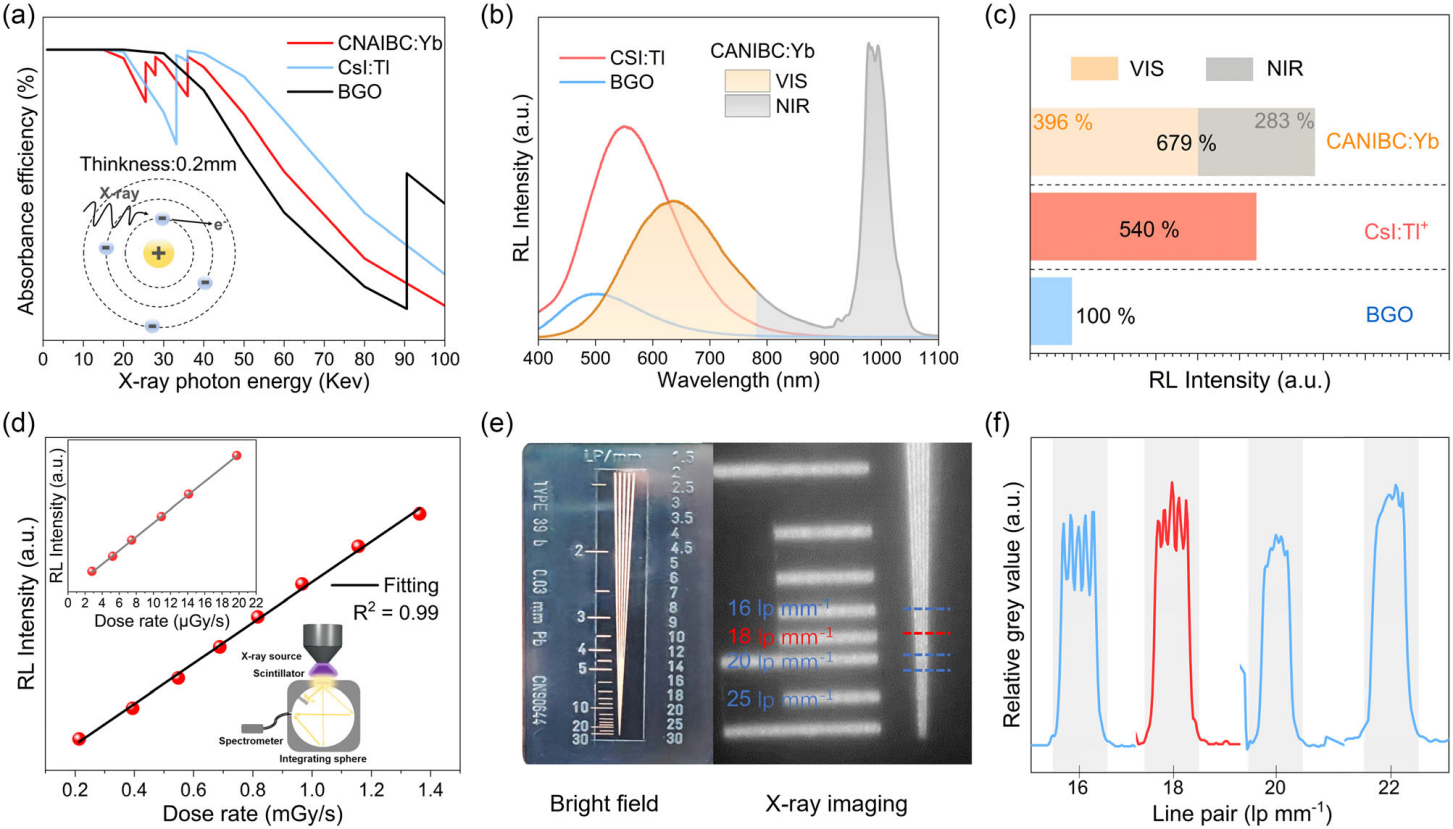
a) Attenuation efficiency of CANIBC:Yb, CsI:Tl^+^, and BGO as a function of photon energy, b) RL spectrum (dose rate: 200 μGy s^−1^, voltage: 40 kV), and c) relative LO. d) Variation of RL intensity of CANIBC:Yb with X‐ray dose rate. The inset presents a schematic of the measurement setup. e) The physical image of a standard line pair resolution test chart and the X‐ray imaging results obtained using the CANIBC:Yb thin film. f) The gray value contour of the line pair extracted from (e).

We calculated the X‐ray absorption coefficients of the CANIBC:Yb and typical scintillators (Bi_4_Ge_3_O_12_ and CsI:Tl^+^) in a broad range of photon energies based on the photon cross‐section database (**Figure** **3**a). CANIBC:Yb possesses a high density, thus making its absorption rate in the medical digital radiography region (i.e., 18–30 keV) slightly comparable to that of CsI:Tl^+^ and Bi_4_Ge_3_O_12_ (BGO). LO was calculated for the CANIBC:Yb scintillator using a commercial BGO scintillator as a reference (the detailed calculation process is provided in Supporting Information). Figure 3b depicts the RL spectra of CANIBC:Yb, BGO, and CsI:Tl^+^ scintillators, and the corresponding LOs were derived from the integral areas of these spectra.^[^ ^25^ ^]^ As shown in Figure 3c, the LO of CANIBC:Yb is 6.79 times higher than that of BGO scintillators, with 2.83 times higher in the NIR region. The integral RL intensity of the CANIBC:Yb as a function of incident X‐ray dose rate is presented in Figure 3d. The significant linear fitting relationship reveals the exceptional responsiveness of CANIBC:Yb to X‐ray, indicating its broad potential for application in the field of X‐ray detection.^[^ ^26^ ^]^

Subsequently, a comprehensive study was conducted to systematically evaluate the stability of CANIBC:Yb under environmental conditions and persistent X‐ray irradiation. The XRD patterns of CANIBC:Yb did not exhibit any spurious peaks when exposed to air for a long period of time, indicating a high degree of structural stability (Figure S16, Supporting Information). As shown in Figure S17, Supporting Information, under a dose rate of 8 mGy s^−1^, the RL intensity remained stable throughout 60 consecutive cycles of on–off X‐ray irradiation. Remarkably, even under continuous X‐ray irradiation at a high dose rate of 8 mGy s^−1^ for 1 h, the RL intensity of CANIBC:Yb sustained its stability. It demonstrates the long‐term stability of CANIBC:Yb in X‐ray imaging applications.

As depicted in Figure S18, Supporting Information, a large‐scale flexible CANIBC:Yb film (100 cm^2^) was fabricated to accommodate irregular biological surfaces. To achieve a more precise measurement of the imaging spatial resolution of the corresponding film, X‐ray imaging was performed using a Nikon D500 camera with high spatial resolution (>25 lp mm^−1^) (Figure S19, Supporting Information). Employed the X‐ray imaging system, nondestructive inspections of intricate chips (Figure S20, Supporting Information) could be conducted. As shown in Figure 3e,f, the spatial resolution of the CANIBC:Yb film recorded using a standard line pair gauge exceeds 18 lp mm^−1^, which typically exhibits a resolution of 10 lp mm^−1^, superior to that of commercial CsI:Tl^+^ detectors.^[^ ^27^ ^]^ To quantify the spatial resolution, the modulation transfer function (MTF) was calculated using slanted‐edge images. As depicted in Figure S21, Supporting Information, the spatial resolution, defined as the spatial frequency value at MTF = 0.2, is determined to be 18.5 lp mm^−1^, which is consistent with the results obtained from the standard line pair card test. The aforementioned results robustly demonstrate the successful fabrication of high‐quality CANIBC:Yb film.

The currently utilized commercial scintillators predominantly emit light within the visible spectrum (400–780 nm), necessitating the performance of X‐ray imaging under dark conditions to mitigate interference from external light.[^26^, ^28^] Compared with visible light, NIR light boasts advantages such as strong anti‐interference capability, low scattering rate, and high signal‐to‐noise ratio, making it promising for a wide range of applications in biological imaging^[^ ^29^ ^]^ and medical detection.^[^ ^30^ ^]^ Therefore, the utilization of NIR emitting scintillators in X‐ray imaging holds promise as a solution to the aforementioned issues. Subsequently, we conducted X‐ray imaging experiments on CANIBC:Yb thin films under the influence of intense lighting and strong UV light. The image reception devices included CMOS and an InGaAs detectors, and Figure S22, Supporting Information, illustrates the respective response ranges of these two detectors. It is noteworthy that the NIR emission spectrum of CANIBC:Yb exceptionally matches well with the response range of InGaAs detectors. The unique characteristic substantially benefits the capture of the produced NIR photons, effectively circumventing contrast degradation and deviation of signal acquisition. Consequently, it generally promotes the corresponding image quality for biomedical measurement and medical diagnosis.

**Figure 4:**
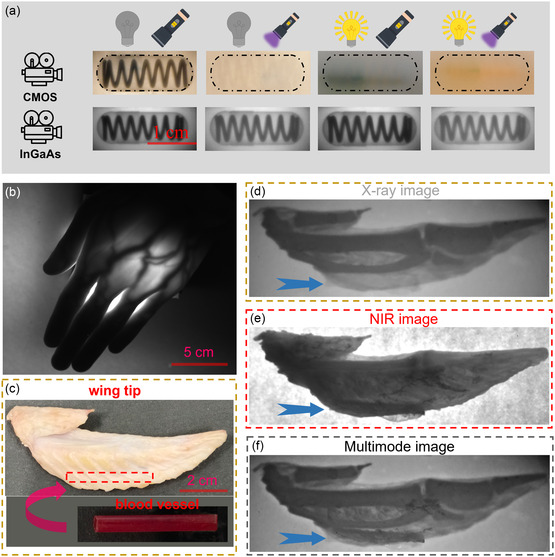
a) X‐ray imaging pictures of the spring inside the capsule were captured using CMOS and InGaAs detectors under different external light interferences, respectively. b) NIR imaging is accomplished using NIR pc‐LEDs. c) Insertion of self‐made blood vessels into the wing tips of edible chicken. d) X‐ray imaging of the chicken wing tips, e) NIR imaging, and f) multimode imaging.

As depicted in **Figure** **4**a, S23, Supporting Information, using a CMOS detector, the CANIBC:Yb film failed to achieve clear imaging due to the intense lighting and UV light interference. In a stark contrast, when utilizing an InGaAs detector, the CANIBC:Yb film was able to produce distinct imaging results even under the combined influence of both lighting and UV light. Furthermore, the CANIBC:Yb film retained its high‐quality X‐ray imaging capability even in complex environments such as high temperatures and underwater conditions (Figure S24, Supporting Information). These findings validate the feasibility of utilizing NIR scintillators for outdoor and portable multifunctional imaging in complex environments, including ambient light interference, high temperatures, and underwater conditions.

By coupling CANIBC:Yb with a commercial 365 nm UV chip, an NIR phosphor‐converted light‐emitting diodes (pc‐LED) was fabricated and applied to in vitro NIR imaging. As shown in Figure 4b, the use of the NIR pc‐LED for in vitro NIR imaging enables a clear visualization of the blood vessel distribution within the interior of palm. This suggests that CANIBC:Yb perovskites possess immense potential for applications in vitro NIR imaging.

As shown in Figure 4c, to demonstrate the advantages of multifunctional imaging, we inserted a self‐made vessel (the plastic tube is made of polypropylene and the blood is pig blood) into a chicken wing tip and performed X‐ray and in vitro NIR imaging separately (Figure 1). For the X‐ray image, the bone distribution inside the wing tip is clearly presented, owing to the higher density and stronger X‐ray absorption capability of bones (Figure 4d). The NIR image clearly reveals the distribution of blood vessels, resulting from the strong absorption of NIR light by hemoglobin in the blood (Figure 4e). The employment of a single detector for multifunctional imaging eliminates the issues of differing resolutions and signal‐to‐noise ratios between two separate images. As depicted in Figure 4f, by combining the two images using optimized weighting coefficients, a multifunctional imaging map is obtained, which facilitates a comprehensive analysis of the internal details of the chicken wing tip. It underscores the significant potential of this technology for future applications in clinical medicine.

## 3 Conclusion

In summary, CANIBC:Yb, featuring efficient NIR luminescence excited by X‐ray and UV light, has been explored. The superior NIR emission aligns with the response range of InGaAs detectors, enabling high‐quality multifunctional imaging. Moreover, co‐alloying strategy of Na^+^ and Bi^3+^ effectively modifies the local structural symmetry of CAIC:Yb, breaking the parity‐forbidden transition, and reducing the electron–phonon coupling strength, which results in a marked enhancement in both PL and RL intensities. Leveraging the robust anti‐interference properties of NIR light, high‐quality X‐ray imaging is achieved even in intense lighting and UV light environments, paving the way for the development of outdoor and portable imaging devices. This work unequivocally demonstrates a multifunctional bioimaging with the explored NIR materials via a single‐detector showcasing a complete medical atlas for biomedical measurement and medical diagnosis.

## 4 Experimental Section

4.1

4.1.1

##### Materials

Cesium chloride (CsCl, 99.9%), silver chloride (AgCl, 99.9%), sodium chloride (NaCl, 99.9%), indium chloride (InCl_3_, 99.9%), bismuth chloride (BiCl_3_, 99.9%), ytterbium chloride hexahydrate (YbCl_3_·6H_2_O, 99.99%), erbium chloride hexahydrate (ErCl_3_·6H_2_O, 99.99%), samarium chloride hexahydrate (TmCl_3_·6H_2_O, 99.99%), neodymium chloride hexahydrate (NdCl_3_·6H_2_O, 99.99%), and holmium chloride hexahydrate (HoCl_3_·6H_2_O, 99.9%) were purchased from Aladdin. Hydrochloric acid (HCl, 37 wt%) was purchased from Sinopharm Chemical Reagent Co., Ltd, China. Polydimethylsiloxane (PDMS) was purchased from Dow Corning 184 optical adhesives, including essential components and curing agent. All of the raw materials were used as received.

##### Synthesis of CAIC Single Crystals

High‐quality CAIC single crystal was synthesized by an improved solvothermal method. In a typical synthesis, 2 mmol of CsCl, 1 mmol of AgCl, and 1 mmol of InCl_3_ were dissolved in 12 mL of HCl solution in a 50 mL Teflon autoclave and heated in a stainless‐steel parr autoclave at 180 °C for 12 h, followed by natural cooling to 30 °C. The obtained precipitates were filtered out, washed three times with ethanol, dried in an oven at 70 °C, and then stored in a glass vial under ambient conditions for further characterization.

##### Synthesis of CAIC:Ln (Ln = Yb^3+^, Tm^3+^, Er^3+^, Nd^3+^, and Ho^3+^) Single Crystals

The synthesis process was identical to that of CAIC single crystals, with an additional 2 mmol of LnCl_3_·6H_2_O added.

##### Synthesis of CANIBC:Yb Single Crystals

In a typical synthesis, 2 mmol of CsCl, 0.6 mmol of AgCl, 0.4 mmol of NaCl, 0.85 mmol of InCl_3_, 0.15 mmol of BiCl_3_, and 2 mmol of YbCl_3_·6H_2_O were dissolved in 12 mL of HCl solution in a 50 mL Teflon autoclave and heated in a stainless‐steel parr autoclave at 180 °C for 12 h, followed by natural cooling to 30 °C. The obtained precipitates were filtered out, washed three times with ethanol, dried in an oven at 70 °C, and then stored in a glass vial under ambient conditions for further characterization.

##### Synthesis of CANIBC:Yb@PDMS Flexible Film

The CANIBC:Yb single crystal was manually ground in a ceramic mortar for 30 min to achieve initial refinement. Then, the CANIBC:Yb powder was repeatedly sifted through a 500‐mesh sieve to obtain ultrafine particles with uniform size. The PDMS was prepared by mixing the prepolymer and curing agent at a volume ratio of 10:1. CANIBC:Yb powder was mixed with PDMS (1:2 mass ratio) and stirred well and left to stand under vacuum for 30 min. The mixture was poured onto a glass slide for initial diffusion. Subsequently, it was placed in a vacuum drying oven to achieve complete diffusion. Finally, the vacuum drying oven was set to 100 °C and heated for 5 h to obtain the CANIBC:Yb@PDMS flexible film.

##### Preparation of NIR pc‐LED Devices

The NIR pc‐LED devices were prepared by mixing CANIBC:Yb with epoxy resin according to the mass ratio of 1:2 and integrating them uniformly with a 365 nm UV chip.

##### Statistical Analysis: Preprocessing of Data

XRD (Figure S1b, S14, and S16, Supporting Information), absorption spectra (Figure S6, Supporting Information), and Raman spectra (Figure S12, Supporting Information) data were normalized to better present the data content. The STE luminescence peaks of PL and RL spectra in Figure 2f and S15, Supporting Information, were normalized.

## Conflict of Interest

The authors declare no conflict of interest.

## Author Contributions

**Tianchi Wang**: methodology (lead); validation (lead); visualization (lead); and writing—original draft (lead). **Jiabo Sun**: validation (equal) and visualization (equal). **Zhaowei Teng**: investigation (lead). **Shuyi Yao**: validation (equal) and writing—review and editing (equal). **Junheng Yuan:** validation (equal). **Lulu Han**: validation (equal). **Dedan Mu**: validation (equal). **Hao Song**: validation (equal). **Xue Yu**: writing—review and editing (lead). **Xuhui Xu**: conceptualization (lead); resources (lead); and supervision (lead).

## Supporting information

Supplementary Material

## Data Availability

The data that support the findings of this study are available on request from the corresponding author. The data are not publicly available due to privacy or ethical restrictions.
