# Supplementary material for: Near‐Infrared Emission Perovskites for Multifunctional Bioimaging
Source: Small Sci. 2025 Feb 21;5(6):2500033. doi: 10.1002/smsc.202500033 (PMC12168608; doi:10.1002/smsc.202500033)
Supplement: Supplementary file 1 — Supplementary Material [file SMSC-5-2500033-s001.pdf]

Supporting Information

Near-Infrared Emission Perovskites for Multifunctional Bioimaging

Tianchi Wang, Jiabo Sun, Zhaowei Teng, Shuyi Yao, Junheng Yuan, Lulu Han, Dedan Mu, Hao Song, Xue Yu*, Xuhui Xu**

T. Wang, Z. Teng, X. Xu

The Central Laboratory and Department of Orthopedic, The Second Affiliated Hospital of Kunming Medical University, Kunming 650106, Yunnan, P. R. China.

E-mails: xuxuh07@126.com; tengzhaowei2002@163.com

T. Wang, J. Sun, S. Yao, J. Yuan, L. Han, D Mu, H. Song, X. Xu

Faculty of Materials Science and Engineering, Key Laboratory of Advanced Materials of Yunnan Province, Kunming University of Science and Technology, Kunming 650093, Yunnan, P. R. China.

X. Yu

School of Mechanical Engineering, Institute for Advanced Materials Deformation and Damage from Multi-Scale, Chengdu University, Chengdu 610106, Sichuan, P. R. China.

E-mails: yuyu6593@126.com;

Supplementary Tables

Table S1. The doping concentrations of samples CAIC: Yb, CAIBC: Yb and CANIBC: Yb obtained from ICP-MS data.

Table S2. Elemental percentage (mol%) determined from EDX data for the CANIBC: Yb sample.

Table S3. S value of different compounds and PLQY summary table.

Supplementary Figures

Figure S1. Schematic representation of the crystal structure of CAIC host and CAIC with Na^{+}, Bi^{3+} and Yb^{3+}.

Figure S2. SEM images of CAIC, CAIC:Yb, CAIBC:Yb, CANIBC:Yb crystals and the corresponding elemental mapping images.

Figure S3. EDS spectrum of CANIBC:Yb.

Figure S4. XPS spectra of CAIC, CAIC:Yb, CAIBC:Yb and CANIBC:Yb.

Figure S5. The ratio of STE luminescence intensity of CAIC, CAIC:Yb, CAIBC:Yb, and CANIBC:Yb samples under UV and X-ray excitation.

Figure S6. Absorption spectra, Tauc plots of CAIC, CAIC:Yb, CAIBC:Yb and CANIBC:Yb samples, respectively.

Figure S7. Band structure and Total and partial density of states for CAIC and CANIBC.

Figure S8. (a) PLE spectra of CAIC, CAIC:Yb, CAIBC:Yb and CANIBC:Yb samples.

Figure S9. Temperature-dependent PL spectra of CAIC:Yb and CANIBC:Yb samples.

Figure S10. The variation of STE intensity with temperature for CAIC:Yb and CANIBC:Yb.

Figure S11. Corresponding fitting curves of the emission intensity variation with temperature for CAIC:Yb and CANIBC:Yb.

Figure S12. Raman spectra of CAIC:Yb, CAIBC:Yb and CANIBC:Yb samples.

Figure S13. Proposed photophysical processes involving both STE recombination and Yb^{3+} 4f-4f transitions via free exciton sensitization under UV excitation.

Figure S14. XRD patterns of CAIC:Ln (Ln = Tm^{3+}, Er^{3+}, Nd^{3+} and Ho^{3+}).

Figure S15. Comparison of PL and RL spectra of CAIC:Tm, CAIC:Er, CAIC:Nd and CAIC:Ho.

Figure S16. XRD patterns of CANIBC:Yb measured after exposed to ambient air for different days.

Figure S17. CANIBC:Yb is tested with 60 X-ray switching cycles and continuous high-dose X-ray irradiation.

Figure S18. Large-Scale flexible CANIBC:Yb Films.

Figure S19. Schematic diagram of the X-ray imaging system.

Figure S20. Photos of precision chips were captured under sunlight and X-ray exposure.

Figure S21. MTF curve based on CANIBC:Yb thin film.

Figure S22. Response range of COMS and InGaAS detectors.

Figure S23. The specific process of X-ray imaging under intense light and ultraviolet light.

Figure S24. X-ray imaging of CANIBC:Yb thin films captured at 293 and 383 K.

Characterization Section

XRD measurement: The phase of as-explored samples was identified by X-ray powder diffraction (XRD) (D8ADVANCE/Germany Bruker X-ray diffractometer) with Cu-Kα radiation at a scanning step of 0.02° in 2θ range from 20 to 60° operating at 40 kV and 30 mA (Rigaku Model D/max-2200).

PLE and PL spectra: An Edinburgh FLS-980 fluorescence spectrometer was employed to record the photoluminescence (PL), photoluminescence excitation (PLE) spectra.

Time-resolved PL decay spectra: Time-resolved PL (TRPL) decay spectra were collected on an Edinburgh FLS-980 fluorescence spectrometer using a TCSPC module (Pico Harp 300) and a SPAD detector (IDQ, id100).

X-ray photoelectron spectra (XPS): XPS analysis was carried out using a Thermo Fisher Scientific K-Alpha and Al-Kα X-ray source (hν = 1486.6 eV) with a 400 nm spot size and 30 eV pass energy.

Scanning electron microscopy (SEM) characterization: SEM images were captured with a FEI Quanta 650 field emission scanning electron microscope Elemental mapping analyses via STEM-HAADF and STEM-EDS were conducted with an FEI-TecnaiTalos-F2000 (200 kV). The EDS elemental analysis results provide the distribution of Cs, Ag, Na, In, Bi, Yb, and Cl in CANIBC:Yb.

Inductively coupled plasma mass spectroscopy (ICP-MS): The actual chemical compositions were measured by ICP-MS using a Perkins-Elmer Optima 3300DV spectrometer to determine the actual doping concentration of Yb^{3+} in the sample.

Absorption and reflectance spectra: The absorption and reflectance spectra were measured on an ultraviolet-visible spectrophotometer (PerkinElmer Instruments, Lambda 950) with an integrating sphere, which was calibrated by measuring a reference material (MgO powder) simultaneous.

Raman spectra: Raman spectra were recorded by an excitation wavelength of 532 nm using a laser confocal micro-Raman spectroscope. The equipment used is a LabRam HR Evolution model, with a test wave number range of 100-4000 cm^{-1} and a test resolution of 1 cm^{-1}.

X-Ray Characterization: A D8 Focus diffractometer (Bruker) with Cu-Ka radiation (λ = 0.15405 nm) as well as a commercially available and miniaturized X-ray tube (Amptek) with a maximum output of 1200 W were employed for X-ray detection. A range of X-ray dose rate from 50 nGy s^{-1} to 8 mGy s^{-1}) was applied by adjusting the current and voltage of the X-ray source. During measurement, the stability under X-ray irradiation, the X-ray tube voltage was kept being 40 kV and 30 mA with a dose rate of 8 mGy s^{-1}. The distance between the X-ray source and the X-ray photodetector device was about 3 cm. However, the X-ray-induced luminescence photos were taken with a digital camera (Nikon D500 with AF-S Micro-Nikkor 105 mm f/2.8G IF-ED). In order to extinguish the negative influence induced by direct radiation from X-ray source to camera, a reflector was used to deflect the optical path by 90 degrees.

RL spectra: The corresponding RL spectra were recorded using a USB2000 + XR1-ES fiber-coupled fluorescence spectrometer, which was carefully calibrated by a marine optical engineer prior to use. The testing wavelength range is determined to be 400-1150 nm.

Near infrared X-ray imaging: To conduct near-infrared X-ray imaging tests, a

high-performance short-wave infrared camera, model ZephIR 1.7-S, was selected, with a detection range of 900-1800 nm. Considering the effect of the focal spot of the X-ray source on imaging resolution, the X-ray tube was placed far enough away from the scintillation screen to act as a quasi-parallel light source.

Estimation of the steady state X-ray light output (LO):

Using the BGO scintillator as a reference (with its integrated RL intensity set at 100%), the relative RL intensity of CANIBC:Yb was compared. CANIBC:Yb and BGO powders were pressed into wafers with a diameter of 0.6 inches and a thickness of 0.2 mm. CANIBC:Yb and BGO wafer is placed at the same position (to ensure the same distance between scintillator and X-ray tube) in the integrating sphere separately. The RL is collected by an optical fiber and spectrometer (Ocean optics), the LO of CANIBC:Yb can be obtained from the following formula[1]:

$$\frac{LY_{[sample]}}{LY_{BGO}} = \frac{\eta_{BGO} \int I_{sample}(\lambda) d\lambda \times S_{BGO}}{\eta_{sample} \int I_{BGO}(\lambda) d\lambda \times S_{sample}} \quad (1)$$

where η is the X-ray deposited energy percentage of scintillators, *I* is the integrated RL intensity at different wavelengths (λ), S is the irradiation area, and the X-ray dose is assumed to be uniform across the whole scintillator wafer. Through calculation, the absorption efficiency of CANIBC:Yb and BGO wafers with a thickness of 0.2 mm are close to 100% for X-ray photons with an average energy of 8 KeV (when the X-ray tube is bias is 40 kV). Based on this, the LO of CANIBC:Yb wafer scintillators is about 6.79 times that of BGO, with 3.96 times that of BGO in visible light (400-780 nm) and 2.83 times that of BGO in near-infrared light (780-1100 nm). CsI:Tl^{+} scintillator was used as another reference to cross-check the accuracy of this measurement method.

MTF measurement: MTF determines the spatial resolution of imaging system and represents the ability to transfer input signal modulation of spatial frequency relative to its output. A MTF value of 1 indicates the perfect detection of a given spatial frequency. Using slanted-edge method to calculate MTF, we took an X-ray image of a sharp edge

from a piece of lead (thickness: about 0.3 mm). The edge spread function (ESF) was derived by the edge profile, from which we could deduce the line spread function (LSF) by calculating derivative. Finally, the Fourier transform of the LSF defines the MTF, meaning the MTF curves could be calculated by the following formula[2]:

$$MTF(\nu) = F\big(LSF(x)\big) = F\frac{dESF(x)}{dx} \tag{2}$$

Where the ν is spatial frequency, x is the position of pixels. Due to using different optical system, the position of pixels (x) is defined by following formula:

$$x = \frac{N\,d}{\beta} \tag{3}$$

Where the N is the ordinal number of pixels in X-ray edge image, d is the pixel size (11 μm) and β is optical magnification.

First-principles Calculations: The electron structure calculations were performed using the Vienna ab initio simulation package (VASP) based on density functional theory[3]. And Perdew-Burke-Ernzerhof functional under the general gradient approximation (GGA) is used[4]. In the calculations, the experimental structure is directly adopted without any change. The energy cut-off of the plane wave basis is set to 500 eV, and the energy convergence criteria are set to 10^{-5} eV. In the self-consistent electronic minimization, the Brillouin zones are sampled with a 2 × 2 × 2 k-point mesh.

Supporting Tables

Table S1. The doping concentrations of samples CAIC: Yb, CAIBC: Yb and CANIBC: Yb obtained from ICP-MS data. The nominal ratio and the actual doping content are determined based on the equation of [Yb]/[Yb+In].

Materials	Nominal ratio	Actual content
CAIC: Yb	200%	1.41%
CAIBC: Yb	235%	1.93%
CANIBC: Yb	235%	1.96%

Table S2. Elemental percentage (mol%) determined from EDX data for the CANIBC: Yb sample.

Materials	Cs	Ag	Na	In	Bi	Cl	Yb
CANIBC: Yb	21.68%	6.04%	3.86%	8.22%	1.06%	57.93%	1.21%

Table S3. S value of different compounds and PLQY summary table.

Compositions	S value	PLQY	Ref.
CAIC:Yb	82.1	< 1%	this work
$Cs_2NaInCl_6$	80	< 1%	5
$Cs_3Bi_2I_9$	79.5	< 1%	6
$Cs_3Cu_2I_5$	40.33	73.7%	7
CANIBC:Yb	37.9	85%	this work
Rb_2CuBr_3	37.17	98.6	1
Cs_2ZrCl_6	24.29	70%	8

$Rb_7Sb_3Cl_{16}$	9.6	26%	9

Supporting Figures

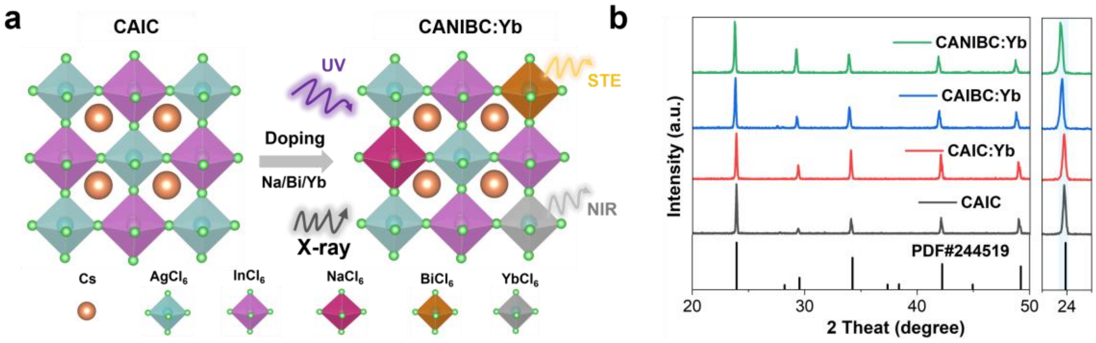

Figure S1. (a) Schematic representation of the crystal structure of CAIC host and CAIC with Na^{+}, Bi^{3+} and Yb^{3+}. (b) XRD patterns of CAIC, CAIC:Yb, CAIBC:Yb and CANIBC:Yb, respectively.

Figure S1a illustrates the crystal structure of CAIC with a highly symmetric face-centered cubic structure (space group: Fm-3m), where $[AgCl_6]^{5-}$ and $[InCl_6]^{3-}$ octahedrons alternately occupy the unit cell and Cs^{+} ions are located in the middle of the cub-octahedral centers. Since Na^{+} (r = 1.02 Å) and Ag^{+} (r = 1.15 Å) have equivalent charges and similar ionic radii, Na^{+} tends to displace Ag^{+} sites to form $[NaCl_6]^{5-}$ octahedra. Bi^{3+} (r = 1.03 Å) and Yb^{3+} (r = 0.86 Å) occupy In^{3+} (r = 0.80 Å) sites to form $[BiCl_6]^{3-}$ and $[YbCl_6]^{3-}$.

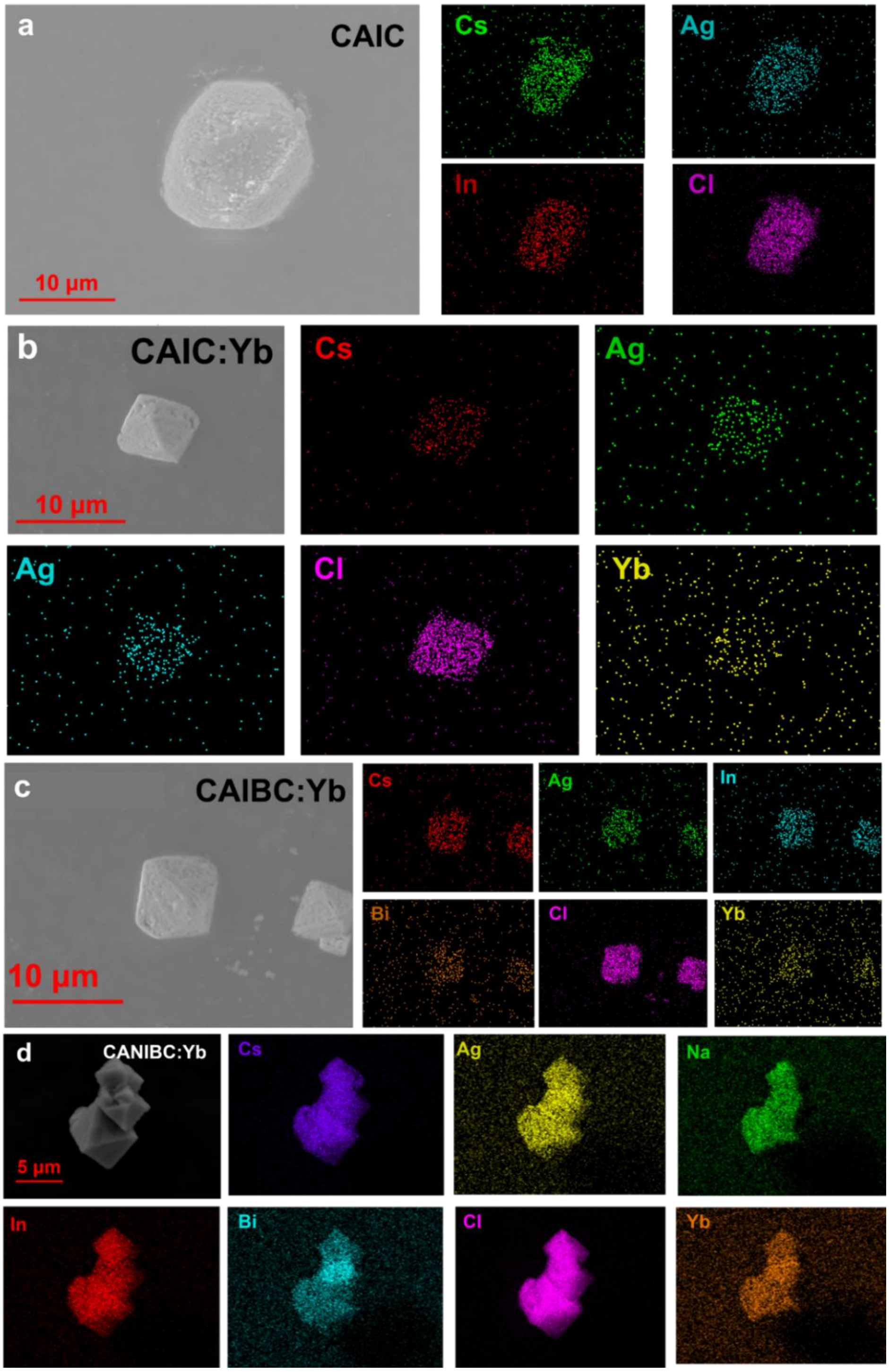

Figure S2. SEM images of (a) CAIC, (b) CAIC:Yb, (c) CAIBC:Yb, (d) CANIBC:Yb crystals and the corresponding elemental mapping images.

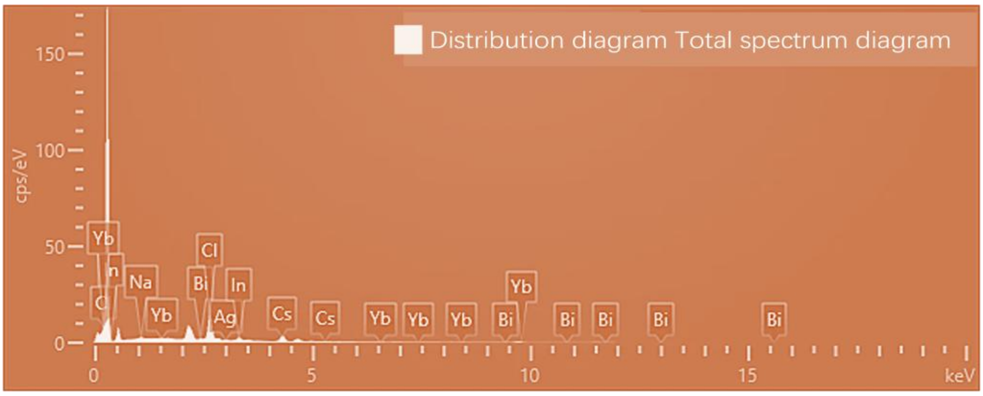

Figure S3. EDS spectrum of CANIBC:Yb.

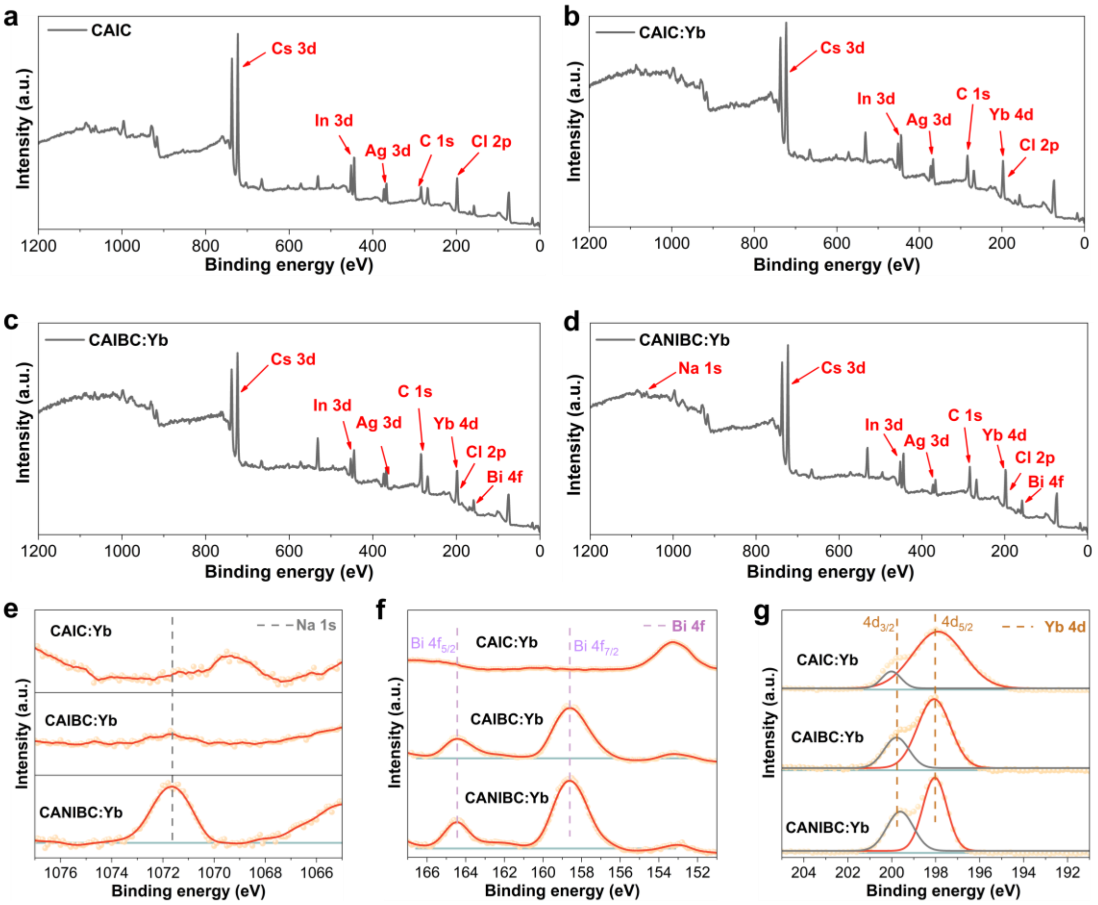

Figure S4. XPS spectra of (a) CAIC, (b) CAIC:Yb, (c) CAIBC:Yb and (d) CANIBC:Yb. High-resolution XPS spectra in the region of (e) Na 1s (f) Bi 4f (g) Yb 4d of the corresponding sample.

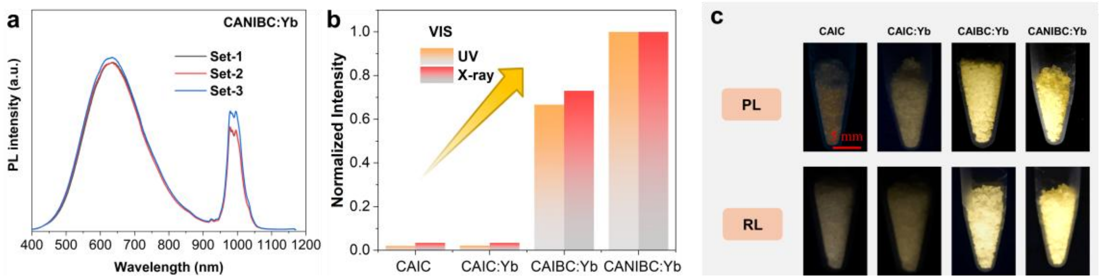

Figure S5. (a) An equal amount of mixed powder (sample + $BaSO_4$) was placed in a quartz sample holder with a certain size of grooves, and the PL spectra of multiple sets of samples were repeatedly tested, named Set-1, Set-2 and Set-3, respectively. (b) The ratio of STE luminescence intensity of CAIC, CAIC:Yb, CAIBC:Yb, and CANIBC:Yb samples under UV and X-ray excitation, and (c) the corresponding luminescence photographs.

Methodology to compare PL Intensity:

The test method was proposed by Nag 's team (Angewandte Chemie International Edition, 2020, 59(28): 11307-11311).[10] For comparison of PL intensity, 5 wt% of the powder sample was thoroughly mixed with $BaSO_4$ powder. $BaSO_4$ does not interfere with excitation and near infrared emission of the sample. So, the role of $BaSO_4$ is to dilute the sample, similar to a solvent in colloidal dispersion. In order to prove the effectiveness of this method for measuring PL intensity, we took an equal amount of mixed (sample + $BaSO_4$) powder into a quartz sample clip with a certain size groove, and repeatedly tested the PL spectra of multiple sets of samples. For example, a mixture of CANIBC: Yb and $BaSO_4$ was prepared and divided into three parts, which were sequentially loaded into the same quartz sample tank with a defined size. It can be clearly seen from figure S5a that the PL intensity of the three groups of samples remains similar. The above experiments show that the mixture of the sample and the $BaSO_4$ powder is quite uniform, so we can compare the PL intensity of two different samples, and the test process completely follows the same procedure.

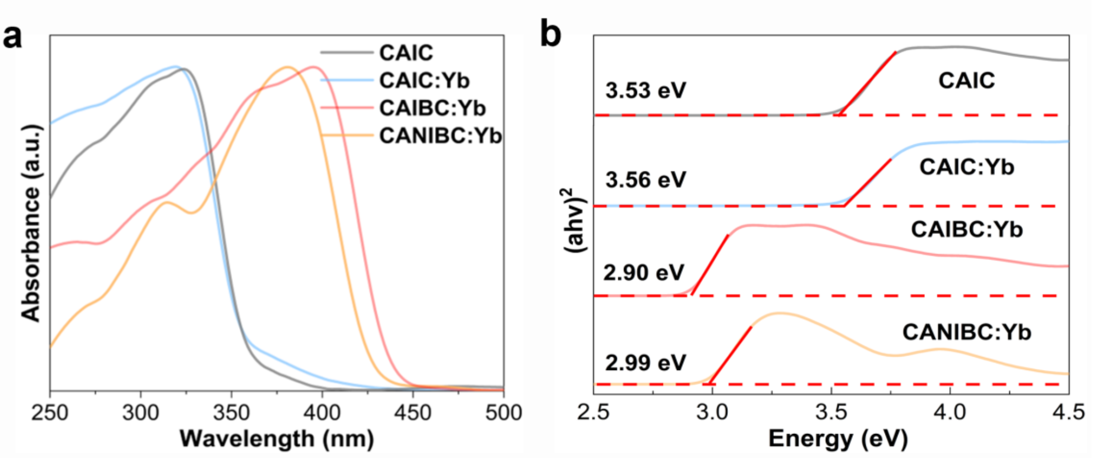

Figure S6. (a) Absorption spectra, (b) Tauc plots of CAIC, CAIC:Yb, CAIBC:Yb and CANIBC:Yb samples, respectively.

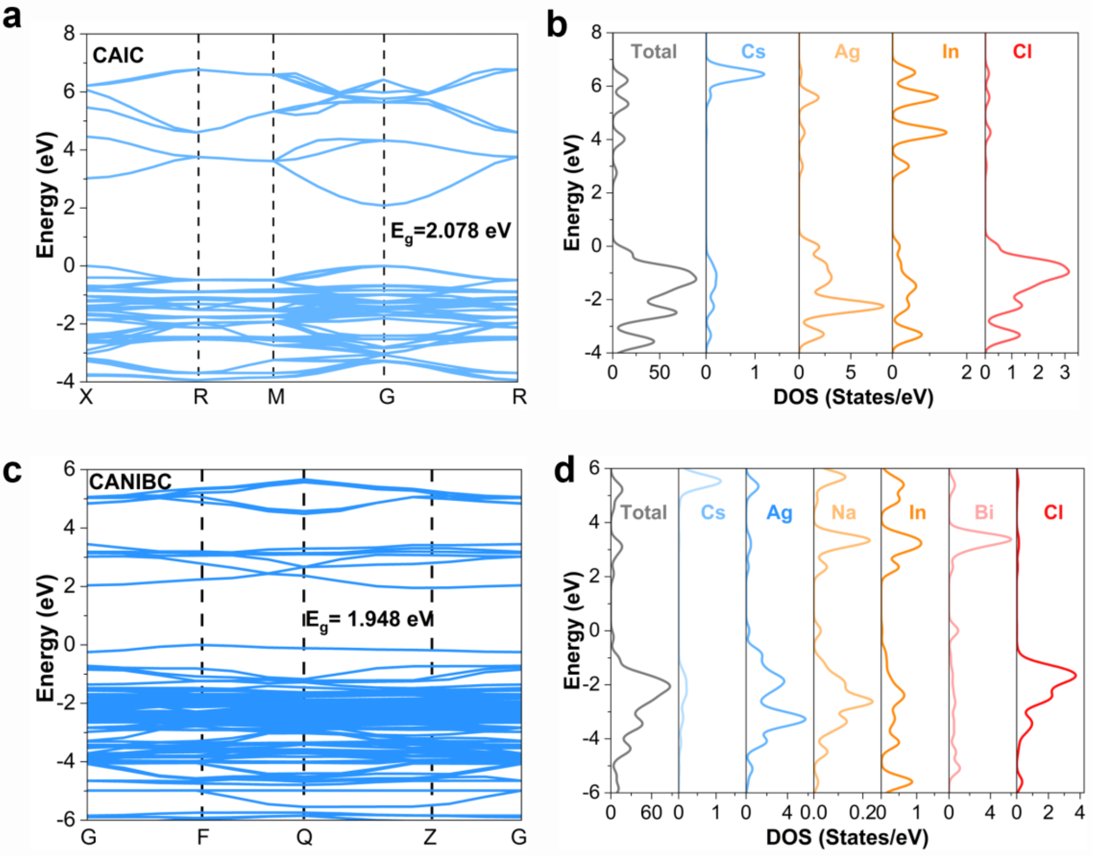

Figure S7. (a, c) Band structure and (b, d) Total and partial density of states for CAIC and CANIBC.

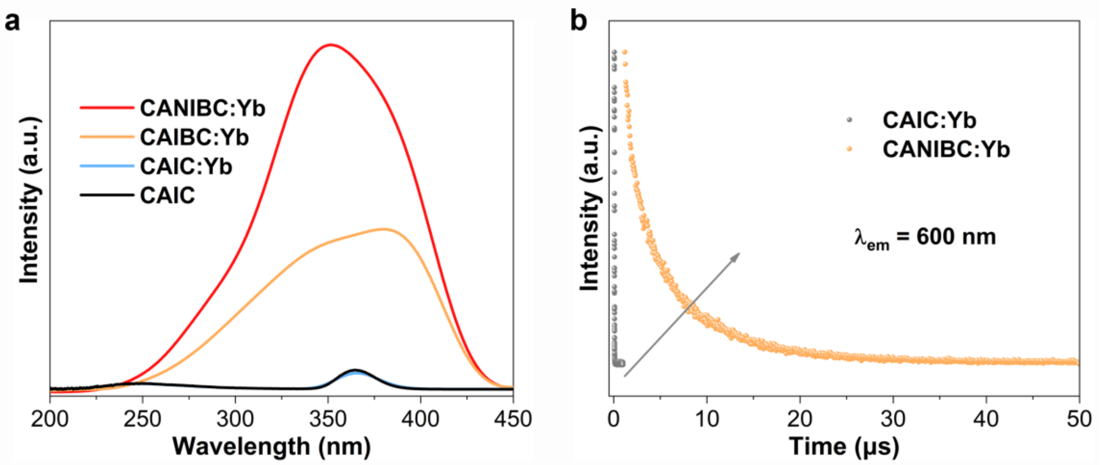

Figure S8. (a) PLE spectra (λ_{em} = 600 nm) of CAIC, CAIC:Yb, CAIBC:Yb and CANIBC:Yb samples. (b) PL decay curves for CAIC:Yb and CANIBC:Yb (λ_{em} = 600 nm), respectively.

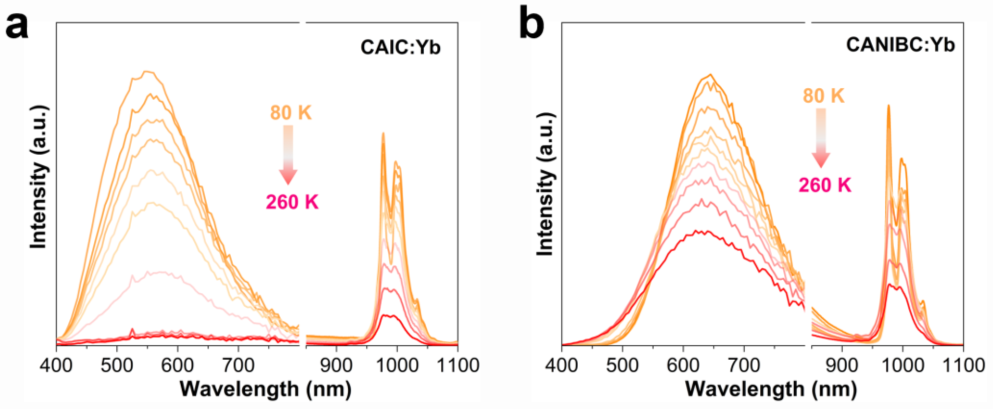

Figure S9. Temperature-dependent (80 ~ 260 K) PL spectra of (a) CAIC:Yb and (b) CANIBC:Yb samples.

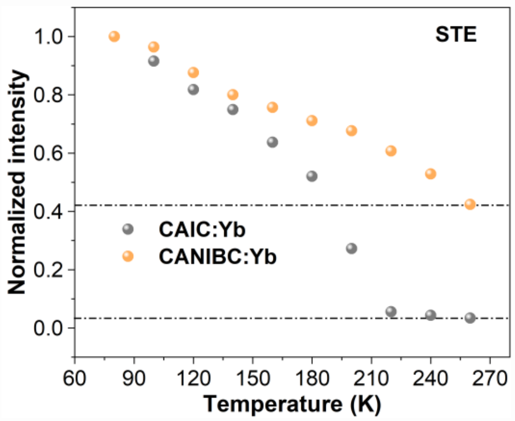

Figure S10. The variation of STE intensity with temperature (80-260 K) for CAIC:Yb and CANIBC:Yb.

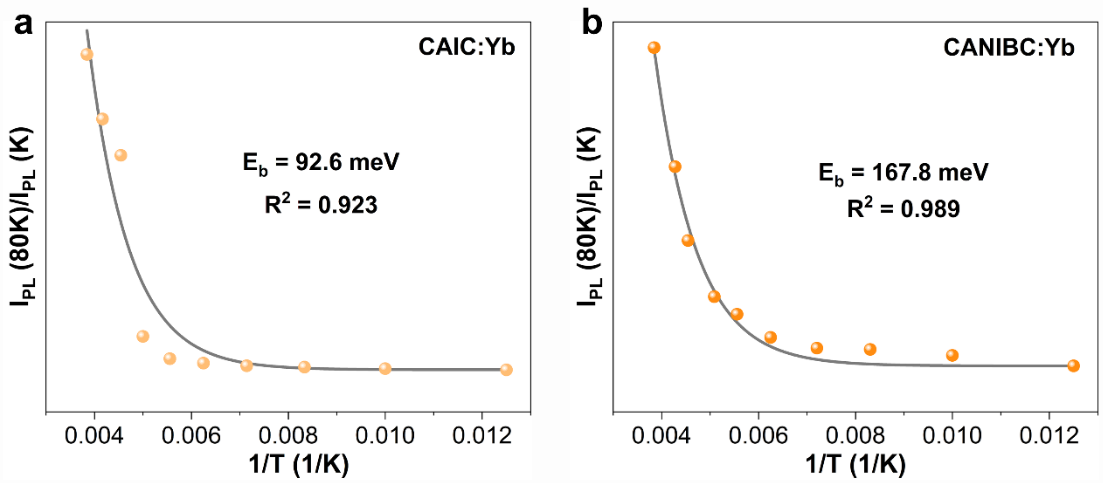

Figure S11. Corresponding fitting curves of the emission intensity variation with temperature for (a) CAIC:Yb and (b) CANIBC:Yb.

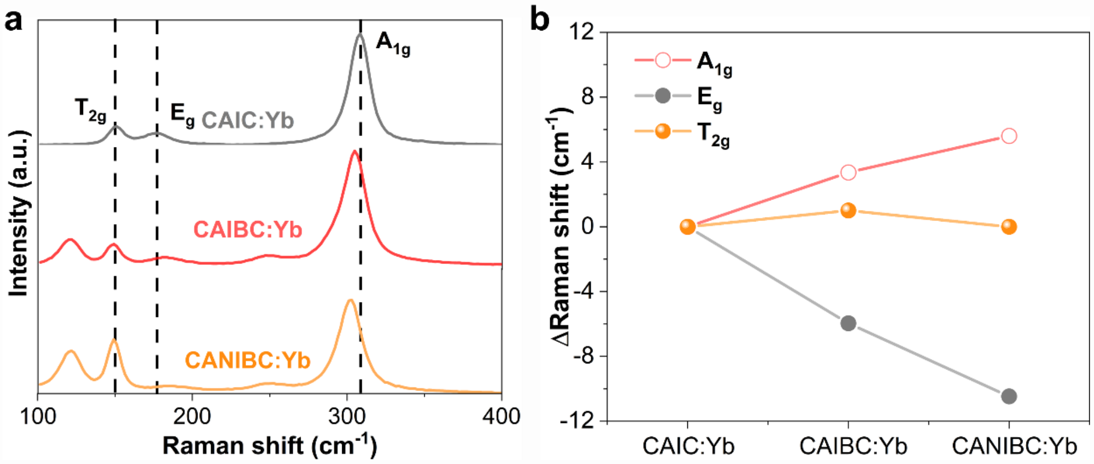

Figure S12. (a) Raman spectra of CAIC:Yb, CAIBC:Yb and CANIBC:Yb samples. (b) Relative Raman peaks shift relative to the respective peak positions of CAIC:Yb.

Raman spectra were measured under excitation by a 532 nm laser diode (Figure S12). The Raman spectra indicate that CAIC:Yb comprises three Raman modes (A_{1g}, E_g, and T_{2g})[11]. A_{1g} and E_g are assigned to symmetric and asymmetric stretching vibrations of $[AgCl_6]^{5-}$ and $[InCl_6]^{3-}$ octahedra, and T_{2g} corresponds to the translation motion of the Cs atom in the cuboctahedra cavity. With the addition of Na^+ and Bi^{3+} dopants, the intensities of A_{1g} and E_g modes weaken owing to the decreased contents of $[AgCl_6]^{5-}$ and $[InCl_6]^{3-}$ octahedrons. Furthermore, the shifting of the Raman signals after Na^+ and Bi^{3+} alloying confirms the occurrence of sub lattice distortion induced by the replacement of $[InCl_6]^{5-}$ and $[AgCl_6]^{3-}$ octahedrons by $[BiCl_6]^{3-}$ and $[NaCl_6]^{5-}$ ones (Figure S12b).

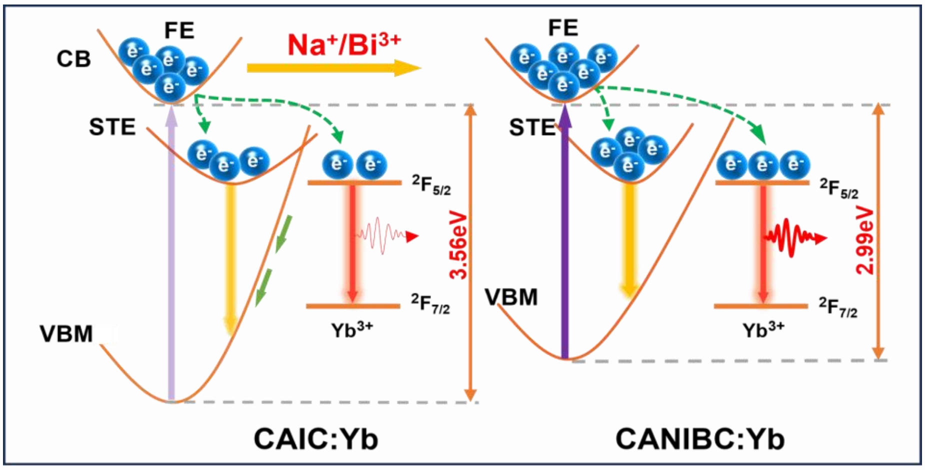

Figure S13. Proposed photophysical processes involving both STE recombination and Yb^{3+} 4f-4f transitions via free exciton sensitization under UV excitation.

Energy transfer occurs from free excitons to both STE and Yb^{3+}, whereas there is no direct energy transfer process between STE and Yb^{3+}. Given that the energy of free excitons originates from the electron transition from the valence band maximum (VBM) to the conduction band minimum (CBM), the luminescence of STE and Yb^{3+} in CAIC:Yb is notably weak due to the presence of odd-even forbidden transitions. Additionally, CAIC:Yb is confronted with strong electron-phonon coupling effects, which gives rise to non-radiative relaxation channels between the excited and ground states, further hindering the luminescence of STE. The dopant of Bi^{3+} significantly enhances the UV absorption capability, overcomes the odd-even forbidden transitions, and effectively inhibits non-radiative relaxation recombination, thereby markedly boosting the luminescence intensity. Additionally, the incorporation of Na^{+} induces the separation and distortion of the $[AgCl_6]^{5-}$ octahedra, reducing the electronic dimensionality, weakening the electron-phonon coupling effect, and further breaking the odd-even forbidden transitions from VBM to CBM. Hence, the co-doping of Na^{+} and Bi^{3+} generates an abundance of free electrons, which can efficiently transfer to both STE and Yb^{3+} and complete the radiative recombination process, thereby enhancing the luminescence efficiency.

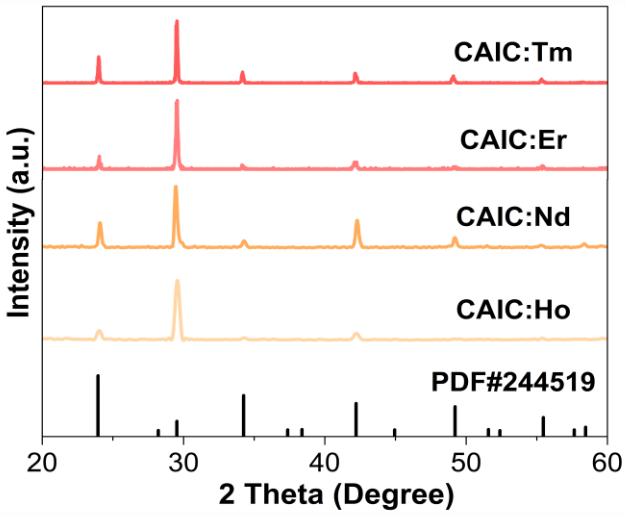

Figure S14. XRD patterns of CAIC:Ln (Ln = Tm^{3+}, Er^{3+}, Nd^{3+} and Ho^{3+}).

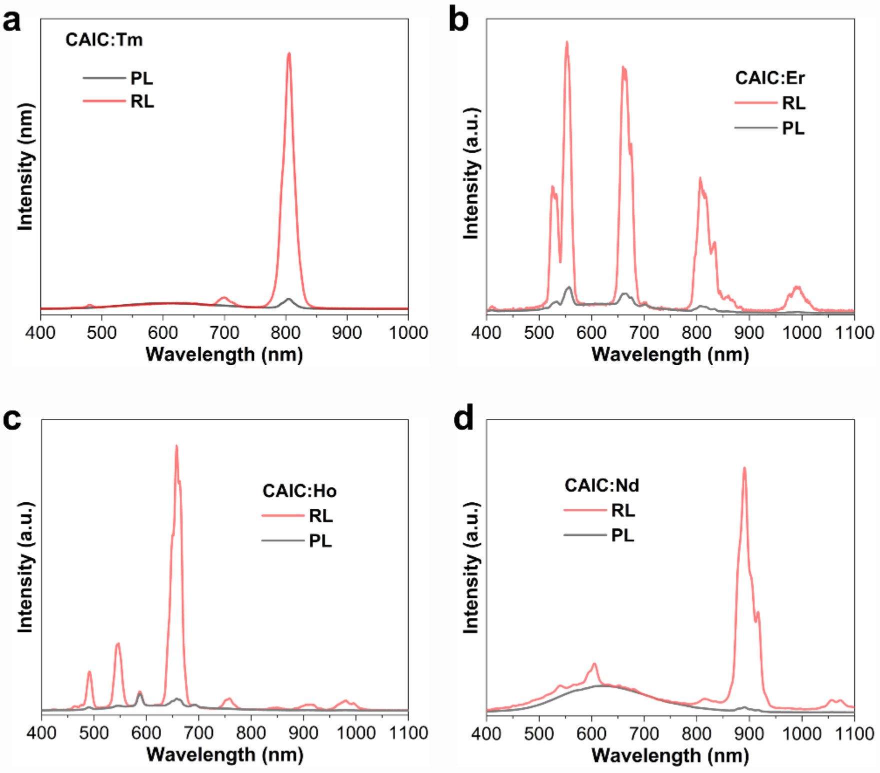

Figure S15. Comparison of PL and RL spectra of (a) CAIC:Tm, (b) CAIC:Er, (c) CAIC:Nd and (d) CAIC:Ho. STE luminescence peaks have been normalized.

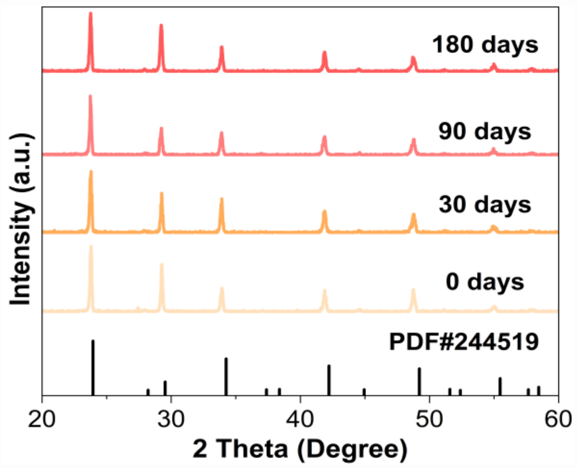

Figure S16. XRD patterns of CANIBC:Yb measured after exposed to ambient air for different days.

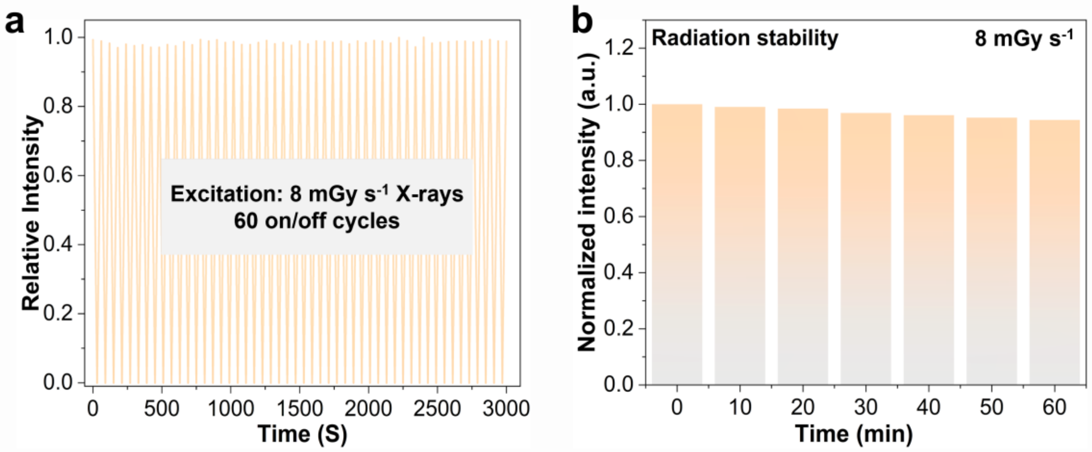

Figure S17. CANIBC:Yb is tested with (a) 60 X-ray switching cycles and (b) continuous high-dose X-ray irradiation.

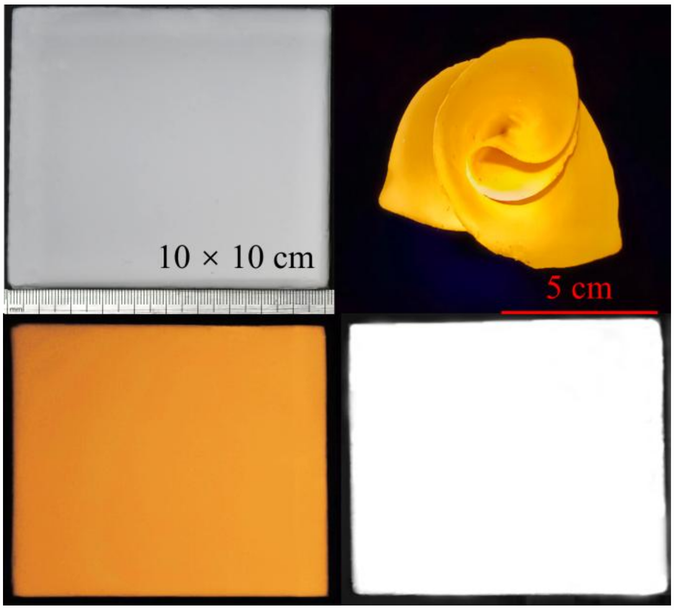

Figure S18. Large-scale flexible CANIBC:Yb films.

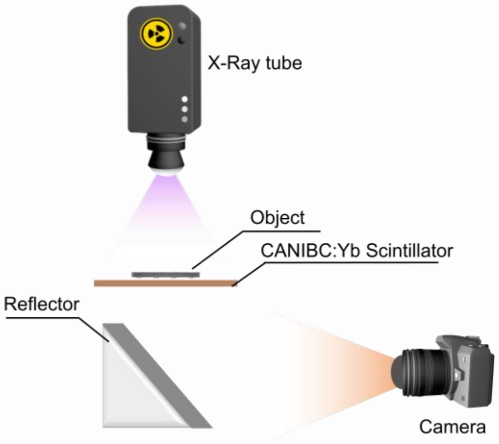

Figure S19. Schematic diagram of the X-ray imaging system.

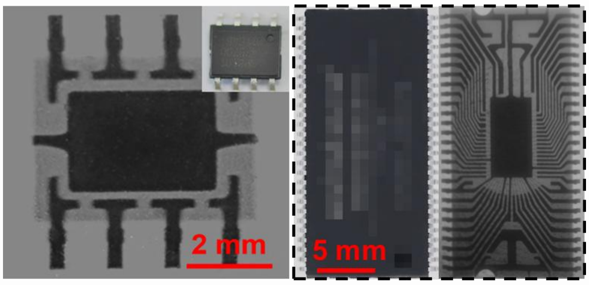

Figure S20. Photos of precision chips were captured under sunlight and X-ray exposure.

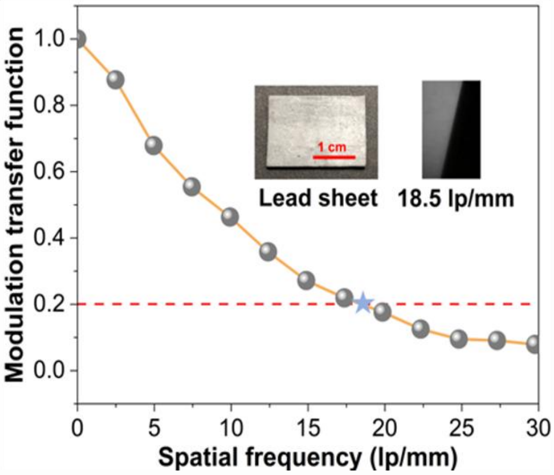

Figure S21. MTF curve based on CANIBC:Yb thin film.

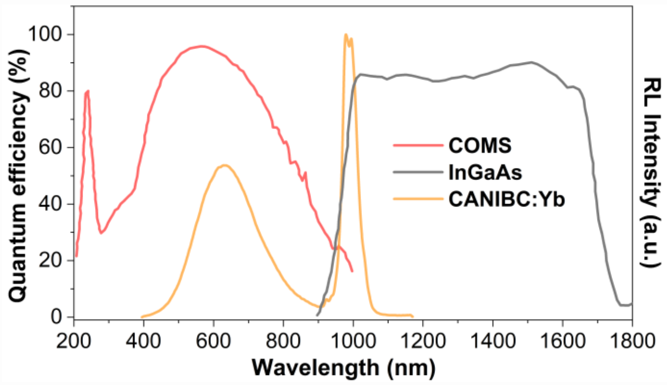

Figure S22. Response range of CMOS and InGaAS detectors.

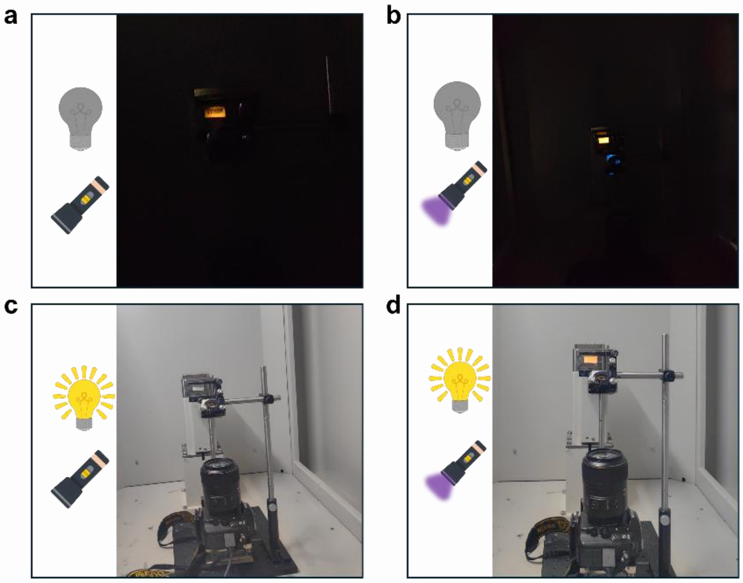

Figure S23. The specific process of X-ray imaging under intense light and ultraviolet light.

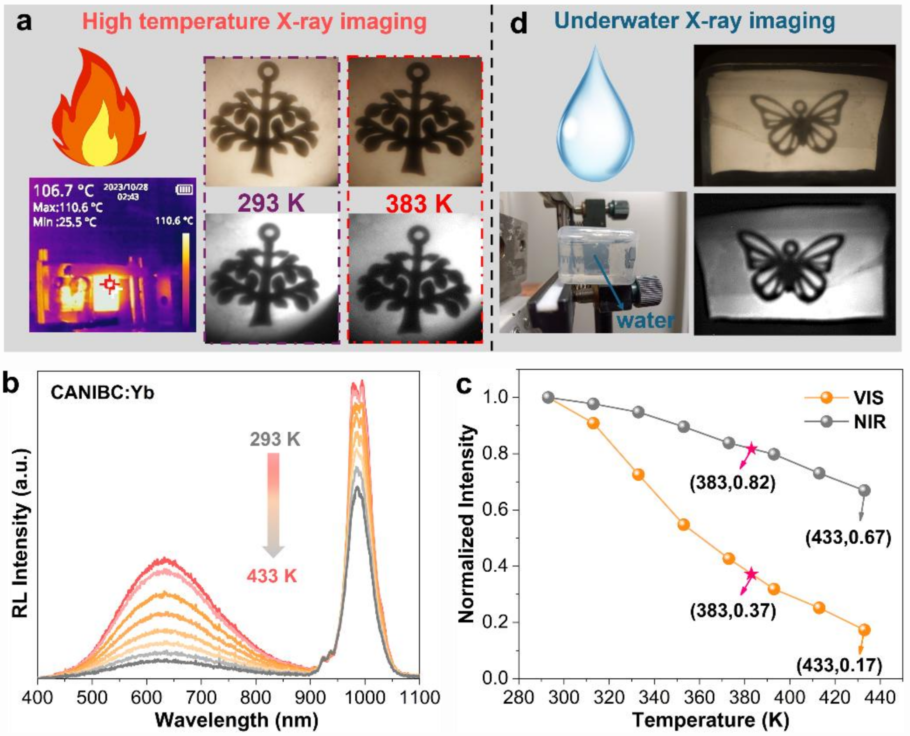

Figure S24. (a) X-ray imaging of CANIBC:Yb thin films captured at 293 and 383 K. (b) Variable temperature spectrogram of CANIBC:Yb. (c) Variable temperature dot-line diagram of CANIBC:Yb. (d) Underwater X-ray imaging of CANIBC:Yb film.

References

[1] B. Yang, L. Yin, G. Niu, J. H. Yuan, K. H. Xue, Z. Tan, X. S. Miao, M. Niu, X. Du, H. Song, *Adv. Mater.* **2019**, *31*, 1904711.

[2] H. Zhang, Z. Yang, M. Zhou, L. Zhao, T. Jiang, H. Yang, X. Yu, J. Qiu, Y. Yang, X. Xu, *Adv. Mater.* **2021**, *33*, 2102529.

[3] G. Kresse, J. Furthmüller, *Comp. Mater. Sci.* **1996**, *6*, 15-50.

[4] a) F. Tran, P. Blaha, *Phys. Rev. Lett.* **2009**, *102*, 226401; b) D. Koller, F. Tran, P. Blaha, *Phys. Rev. B* **2012**, *85*, 155109.

[5] J. Luo, X. Wang, S. Li, J. Liu, Y. Guo, G. Niu, L. Yao, Y. Fu, L. Gao, Q. Dong, *Nature* **2018**, *563*, 541-545.

[6] K. M. McCall, C. C. Stoumpos, S. S. Kostina, M. G. Kanatzidis, B. W. Wessels, *Chem. Mater.* **2017**, *29*, 4129-4145.

[7] L. Lian, M. Zheng, W. Zhang, L. Yin, X. Du, P. Zhang, X. Zhang, J. Gao, D. Zhang, L. Gao, *Advanced Science* **2020**, *7*, 2000195.

[8] F. Zhang, Y. Zhou, Z. Chen, M. Wang, Z. Ma, X. Chen, M. Jia, D. Wu, J. Xiao, X. Li, *Adv. Mater.* **2022**, *34*, 2204801.

[9] Z. Tan, M. Hu, G. Niu, Q. Hu, J. Li, M. Leng, L. Gao, J. Tang, *Sci. Bull.* **2019**, *64*, 904-909.

[10] H. Arfin, J. Kaur, T. Sheikh, S. Chakraborty, A. Nag, *Angew. Chem. Int. Ed.* **2020**, *59*, 11307-11311.

[11] a) X. Cheng, Z. Xie, W. Zheng, R. Li, Z. Deng, D. Tu, X. Shang, J. Xu, Z. Gong, X. Li, *Adv. Sci.* **2022**, *9*, 2103724; b) H. Siddique, Z. Xu, X. Li, S. Saeed, W. Liang, X. Wang, C. Gao, R. Dai, Z. Wang, Z. Zhang, *J. Phys. Chem. Lett.* **2020**, *11*, 9572-9578.
